# Insight on the Void Ratio–Suction Relationship of Compacted Bentonite during Hydration

**DOI:** 10.3390/ma15155173

**Published:** 2022-07-26

**Authors:** Yang Wang, Jun Teng, Qi Huang, Wei Wang, Zhenyang Ren

**Affiliations:** 1 School of Civil and Environmental Engineering, Harbin Institute of Technology (Shenzhen), Shenzhen 518055, China; tengj@hit.edu.cn; 2 Engineering Management Center, Bureau of Public Works of Shenzhen Municipality, Shenzhen 518031, China; huangqi@szwb.gov.cn (Q.H.); thomaswyang@163.com (W.W.); szgwsbsh@163.com (Z.R.)

**Keywords:** compacted bentonite, void ratio–suction curve, swelling-collapse characteristic, swelling index

## Abstract

Investigation on swelling characteristics of buffer/backfill materials during hydration is an important issue in the design of artificial barriers in high-level radioactive waste (HLW) disposal repositories. In this work, for clarifying the characteristic of void ratio–suction relationship for compacted bentonite on hydration path, suction-controlled swelling deformation tests under constant vertical stresses 0.001~40 MPa were carried out on compacted bentonite specimens. Four different types of void ratio-suction curves indicated that swelling-collapse behavior under hydration depends on suction and over-consolidation ratio (OCR), based on which the swelling index was defined. Then, equations were proposed for describing the swelling-collapse characteristic of void ratio–suction curves. Simulation results of suction-controlled swelling deformation tests show that the different types of the hydration deformation curves could be well described by the proposed equations. Obviously, the proposed equations could be used for description and prediction of swelling characteristics of compacted bentonite during hydration, which is also of great importance for the safety assessment of the HLW repositories.

## 1. Introduction

In many countries, compacted bentonite has been selected as the buffer/backfill material for high-level radioactive waste (HLW) disposal [1,2,3,4,5]. During the operation of disposal, compacted bentonite is expected to swell and fill in various gaps and cracks in buffer/backfill materials and surrounding rocks in the repository. The formed barriers could effectively prevent groundwater intrusion from the surrounding geological formations and the possible release of radioactive waste from the canister [6]. Therefore, evaluation and prediction of swelling characteristic of compacted bentonite during hydration is an important issue in the design of artificial barriers in the geological repositories.

Swelling deformation tests under constant stress are generally carried out to investigate the swelling characteristics of compacted bentonite [6,7,8]. Lots of studies [1,9] have reported that the swelling strain is directly affected by the montmorillonite content, dry density, initial water content, and stress applied. Lloret et al. [10] and Zhao et al. [11] reported that, for specimens with a given initial dry density, the swelling strain decreases with increasing stress applied. Meanwhile, for a given stress applied, the swelling strain increases with increasing initial dry density. Dang and Robinet [12] reported that the swelling strain depends on the OCR. Unfortunately, upon the swelling deformation tests in the laboratory, wetting has most often been performed by flooding the soil with water starting from the initial water content to full saturation, without suction control. The relationship between swelling deformation of compacted bentonite with suction during hydration have not been well understood.

For exactly predicting swelling characteristic of compacted bentonite during hydration, several authors [13,14] have tried to predict it by using equations based on the diffuse double layer theories. However, it is difficult to introduce these equations into elastoplastic constitutive models of bentonite for describing volume change behavior on different hydro-mechanical paths. Therefore, for building elastoplastic constitutive models of bentonite, the void ratio–suction relationship of compacted bentonite during hydration also needs to be studied.

In this work, suction-controlled swelling deformation tests under constant vertical stresses were carried out on compacted Gao-Miao-Zi (GMZ) bentonite specimens. The swelling-collapse characteristics under hydration were analyzed. According to the test results, the swelling index was defined. Then, equations were proposed for describing the swelling-collapse characteristics of void ratio–suction curves. Finally, performance of the proposed equations was assessed by simulating suction-controlled swelling deformation tests conducted on compacted specimens of GMZ bentonite.

## 2. Material and Method

### 2.1. Material and Specimen Preparation

**Figure 1:**
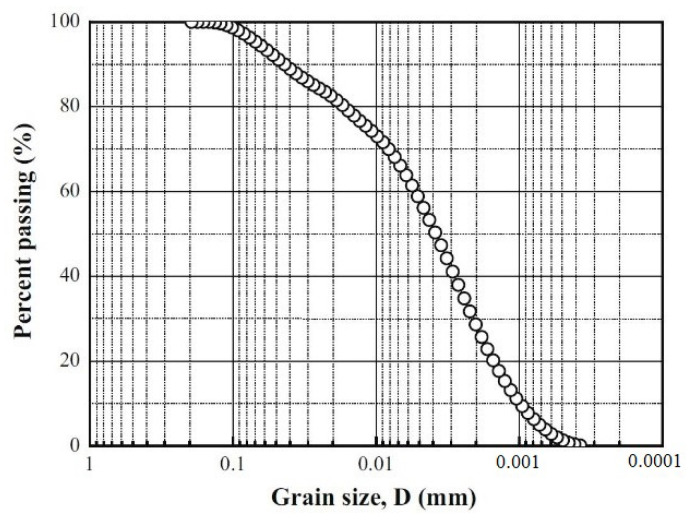
The grain size distribution curve of GMZ bentonite.

GMZ bentonite, a sodium bentonite tested in this work, originated from Inner Mongolia, China [15]. It is composed of more than 75% of montmorillonite with the cation-exchange capacity (CEC) of 77.3 meq/100 g (43.36% Na^+^, 29.14% Ca^2+^, 12.33% Mg^2+^, 2.51% K^+^). The liquid limit and plastic limit are 276% and 37%, respectively, and the specific gravity is 2.67. The grain size distribution curve of GMZ bentonite is shown in Figure 1.

To prepare the specimen, the bentonite powder was firstly equilibrated to suction of 113 MPa (corresponding to an initial water content of 10.2%) using the vapor equilibrium technique [16]. Then, a given quantity of powder was poured into the compaction cell and statically compacted to a target dimension (50 mm in diameter and 10 mm in height) and dry density (1.70 g/cm^3^). The compaction load was statically applied with a displacement rate of 0.1 mm/min. After the target state was reached, the vertical static load was kept for an hour for specimen homogenization.

### 2.2. Experimental Apparatus

**Figure 2:**
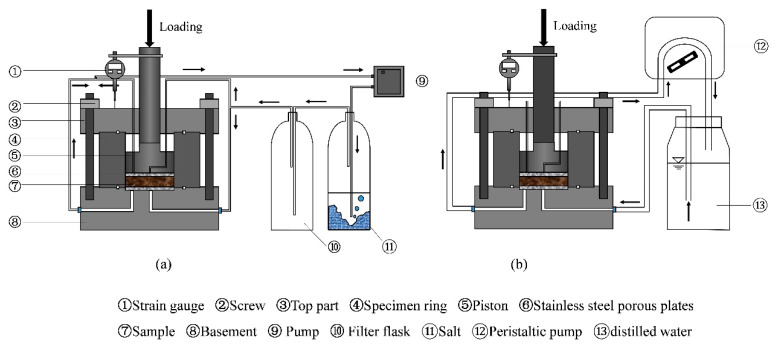
Experimental setup for the suction-control swelling deformation tests. (**a**) Vapor phase technique, (**b**) Water circulation technique.

The experimental apparatus employed for conducting suction-control swelling deformation tests in this work is shown in Figure 2. It included an oedometer cell, a high-pressure load frame, and suction-control systems. The oedometer cell was made up of a basement with a porous plate and a drainage system for water/air circulation, a specimen ring (50 mm in internal diameter) to prevent radial swelling, and a loading piston with two outlets for air circulation. The high-pressure load frame [15] was used for vertical stress applied and a strain gauge (precision of 0.001 mm) was fixed to record the vertical strain.

The suction-control systems as in Figure 2 were used for hydration. The vapor phase method and water circulation method were employed for controlling unsaturated and saturated states, respectively. For applying the vapor phase technique [16,17], vapor of saturated salt solution was circulated in a close system and accelerated by a pump (Figure 2a). Tang and Cui [16] proposed the relationship between the saturated salt solution and its corresponding imposed suction, which was employed in this work. For applying the water circulation technique, the distilled water was circulated through the porous plate at the bottom of the specimen using a peristaltic pump (Figure 2b).

### 2.3. Experimental Procedures

Firstly, to conduct the suction-control swelling deformation test, a specimen (with an initial suction 113 MPa) was installed in the oedemeter cell, sandwiched between two porous stones.

Then, a certain vertical stress (ranging from 0.001 to 40 MPa) was applied and the strain gauge was used to measure the vertical strain of specimen. When the variation of the vertical displacement recorded during 24 h was less than 0.01% [18,19], the corresponding height of the specimen was considered as the initial one.

Afterwards, target suctions were applied step by step using suction-control systems following the path: 113~38~9~4.2~0 MPa. For suctions 38 MPa, 9 MPa, and 4.2 MPa, the vapor of saturated NaCl, KNO_3_, or K_2_SO_4_ solution was circulated in the specimen using the vapor equilibrium technique (as shown in Figure 2a). Finally, for the target suction of 0 MPa (the saturated state) to be controlled, the specimen was infiltrated with distilled water (Figure 2b).

The vertical displacement was monitored. A vertical displacement rate of less than 0.01% per 24 h [18] was adopted as the cut-off criterion of equalization in this paper. When the swelling displacement measured under the current controlling suction was stable, the next suction was applied.

The above-mentioned procedures were repeated on as-prepared specimens under different vertical stresses. In total, 8 tests were performed (Table 1) on specimens under constant vertical loads 0.001~40 MPa.

All the tests were performed at an ambient temperature 25 ± 0.5 °C.

## 3. Results

**Figure 3:**
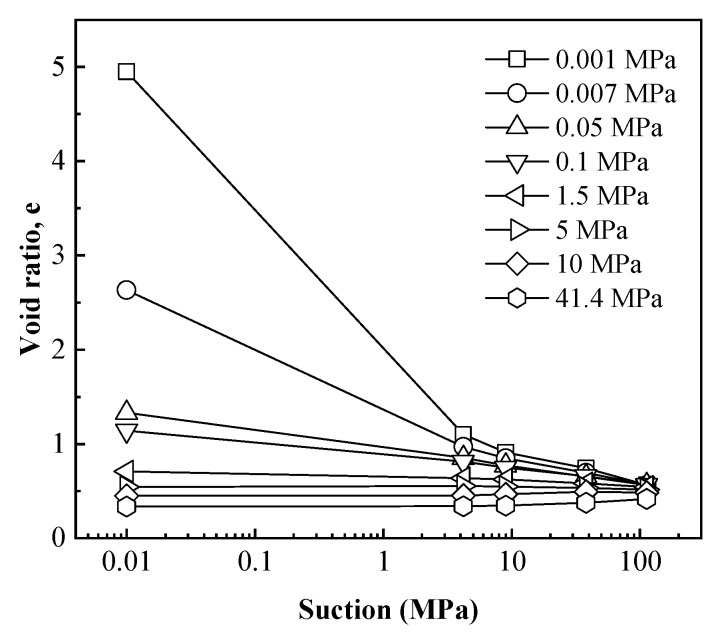
The evolutions of void ratio for specimens with decreasing suction tested under different vertical stresses.

The evolutions of void ratio for specimens with decreasing suction tested under different vertical stresses are plotted in Figure 3. It was observed that the void ratio at every suction decreased significantly with increasing vertical stress, which indicated that swelling deformation of specimens during hydration (suction decrease) processes decrease significantly with increasing vertical stress. As the applied vertical stress increased from 0.001 to 40 MPa, the final value of the void ratio after hydration reduced from 4.95 to 0.34.

**Figure 4:**
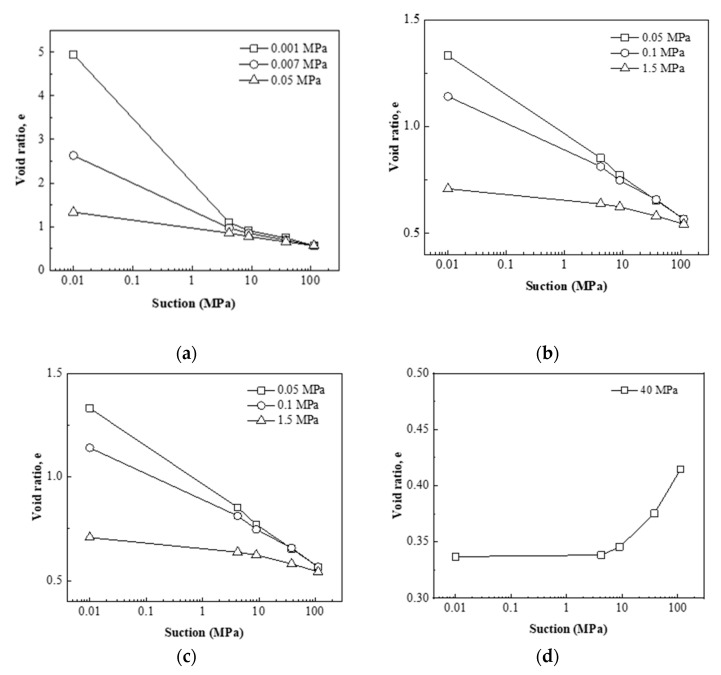
Four typical shapes of the void ratio–suction curves of bentonite under hydration. (**a**) free-swelling type, (**b**) swelling type, (**c**) swelling-collapse type, (**d**) collapse type.

Figure 3 also shows that, different from the common linear-form e-lnp curve, the shapes of e-lns curves were varied, which depended on the applied vertical stress. In summary, the void ratio–suction curves showed four typical shapes, which could be respectively presented in Figure 4.

The first shape of e-lns curves shown in Figure 4a was obtained during hydration under extremely low vertical stress: 0.001, 0.007, and 0.05 MPa. The void ratio gradually increased with decreasing suction, indicating that the bentonite kept producing swelling deformation during the hydration process. In the high suction section (above 4.2 MPa), the e-lns curves were almost linear, while in the low suction section (below 4.2 MPa), the void ratio increased significantly with the decrease of suction. As the vertical stress applied increased, the nonlinearity of the curve gradually decreased. When the stress increased to 0.05 MPa, the curve was almost linear instead of nonlinear. If the stress continued to increase, the curve presented the second shape shown in Figure 4b. The second shape of e-lns curves shown in Figure 4b was obtained during hydration under vertical stress 0.05, 0.1, and 1.5 MPa. The void ratio increased significantly with the decrease of suction; meanwhile, the e-lns curves were almost linear.

**Figure 5:**
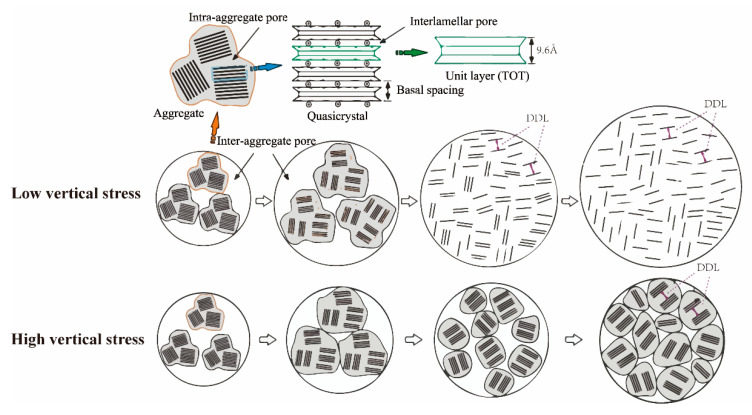
Different swelling processes of bentonite under low and high vertical stresses.

The first and second shape of e-lns curves could be defined as free-swelling and swelling types, respectively. The difference represents the stress-dependent characteristic of the swelling property, which is shown in Figure 5. In case of lower vertical stress (0.001~0.05 MPa), almost without applied stress, the unlimited volume expansion leads to a fully breaking up of particles [20]. The process of fully breaking up particles is the reason for the significantly increasing void ratio in the low-suction section. On the contrary, in case of high vertical stress (0.05~1.5 MPa), with limited volume expansion, a relatively incomplete swelling occurred.

When the stress applied further increased, the third shape of e-lns curves shown in Figure 4c was obtained during hydration under vertical stress of 5 and 10 MPa. The void ratio increased firstly and then decreased with the decrease of suction, indicating that the bentonite produced swelling deformation firstly during the hydration process, and when the suction decreased to a certain value, the soil skeleton collapsed and the soil sample turned to shrinkage deformation. The fourth shape of e-lns curves shown in Figure 4d was obtained during hydration under vertical stress 40 MPa. The void ratio gradually decreased with the decrease of suction, indicating that the skeleton collapses directly after the beginning of the hydration process and the bentonite produces shrinkage deformation directly. The third and fourth shape of e-lns curves could be defined as swelling-collapse and collapse types, respectively.

Actually, it is the different stress path resulting in four shapes of hydration deformation curves. Generally, the loading-collapse (LC)-yielding curve in the s–p plane is defined to judge whether the specimen reaches its yielding state or not in the constitutive model of unsaturated soil [21,22]. The location relationship between the current stress state with the LC yielding curve determines whether the specimen reaches its yielding state or not. When the stress state locates inside the LC yielding curve, the expansive soil generates swelling deformation during hydration. However, as the stress state reaches the LC yielding curve, the expansive soil generates collapse deformation.

**Figure 6:**
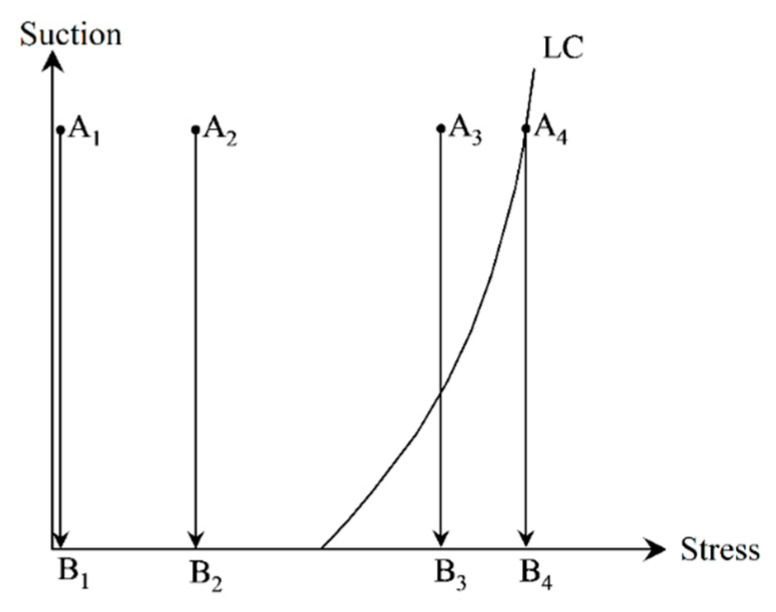
The stress paths of four types of hydration deformation curves. A_1_B_1_: stress path of free-swelling curve, A_2_B_2_: stress path of swelling curve, A_3_B_3_: stress path of swelling-collapse curve, A_4_B_4_: stress path of collapse curve.

The stress paths of four types of hydration deformation curves are presented in Figure 6. As the stress path always moves within the LC yielding curve, the hydration produces the free-swelling and swelling types curves (A_1_B_1_ and A_2_B_2_ in Figure 6). As the stress path firstly moves within the LC yielding curve, then reaches and moves on the LC yielding curve, and the hydration produces the swelling-collapse type curves (A_3_B_3_ in Figure 6). As the stress path always moves on the LC yielding curve, the hydration produces the collapse-type curve (A_4_B_4_ in Figure 6).

## 4. Discussion and Interpretation

**Figure 7:**
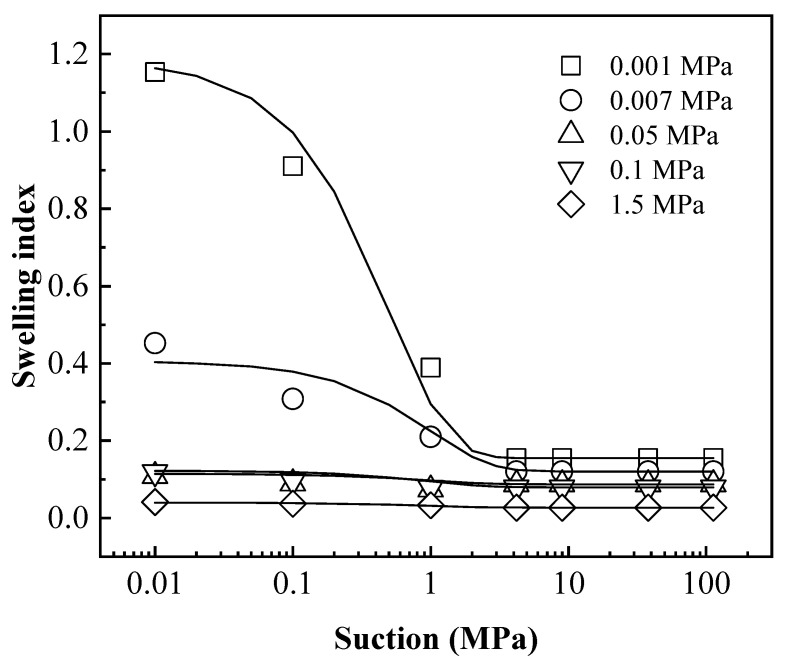
The relationship between the swelling indexes with suction.

In order to quantitatively describe the void ratio–suction (e-lns) relationship during hydration, the tangent slope at each point of e-lns curve was defined as the swelling index. Therefore, the swelling indexes could be obtained based on e-lns curves in Figure 3. The relationship between the swelling indexes with suction for different specimens was shown in Figure 7. It can be found that the swelling index was almost constant in the high-suction section, while it increased significantly in the low-suction section as suction decreased, especially for the free-swelling curve (0.001 and 0.007 MPa). This is mainly due to the fact that at low suction, most montmorillonite crystals in the bentonite specimen could be fully broken into elementary layers due to hydration. Therefore, the following equation is defined to reflect the relationship between the swelling index with suction.

(1) $$\lambda_{s}=A\exp\left(-s\right)+\lambda_{s}^{*}$$

where, $\lambda_{s}$ is the swelling index, *A* is the fitting parameter, and $\lambda_{s}^{*}$ is the initial swelling index in the high-suction section.

**Figure 8:**
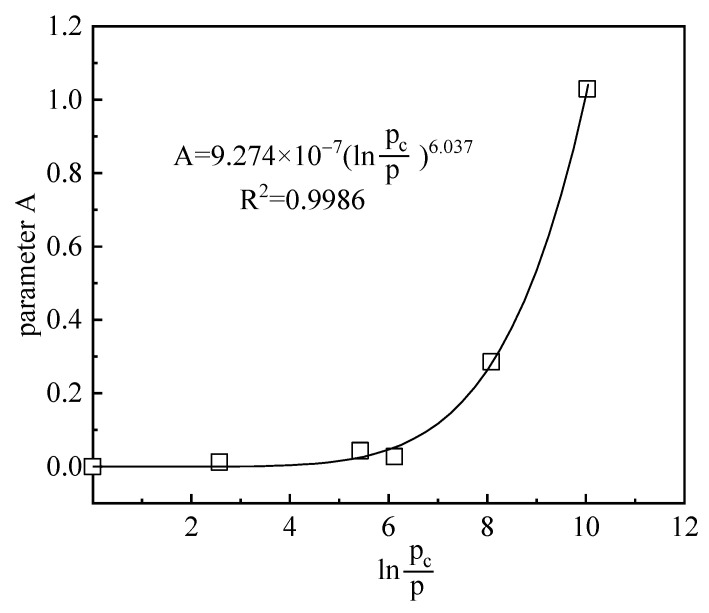
The relationship between the parameter *A* in the swelling index and the over-consolidation ratio (*p*/*p*_0_).

It can be observed in Figure 7 that the nonlinearity of the swelling index with suction changes gradually reduced as the vertical stress increased. For this reason, the relationship between the parameter *A* in the swelling index and the OCR (*p*/*p*_0_) was established as shown in Figure 8. It could be found that the parameter *A* decreased with the gradual decrease of OCR, which indicated that the nonlinearly of swelling index–suction relationship decreases.

**Figure 9:**
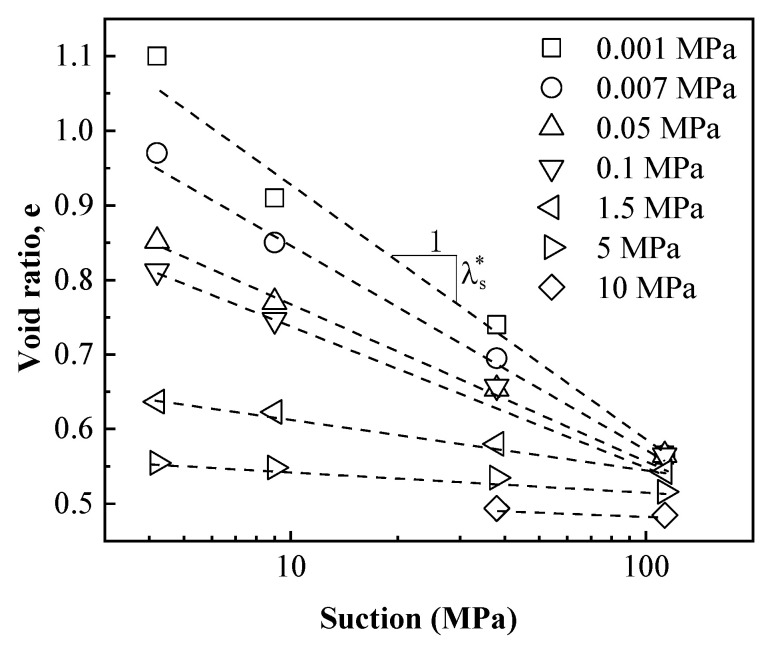
The initial swelling indexes on the free-swelling curves, swelling parts of the swelling curves, and swelling-collapse curves.

**Figure 10:**
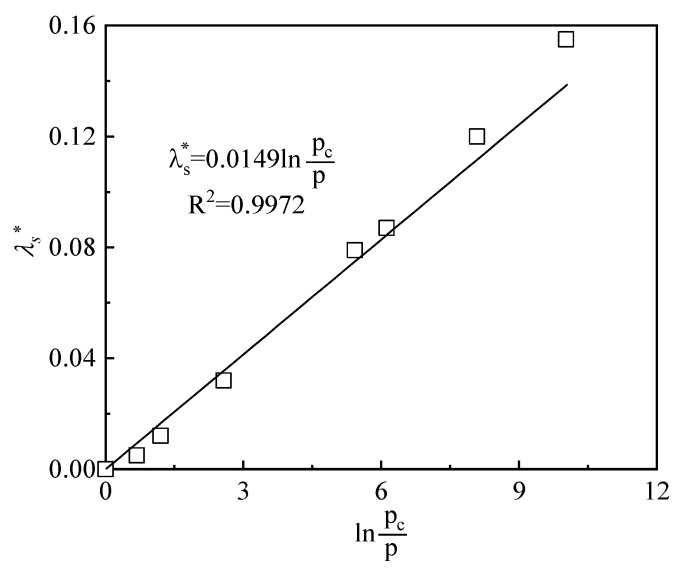
The relationship between the initial swelling index with the over-consolidation ratio (*p*/*p*_0_).

In addition, Figure 9 shows that, for the high-suction section of the free-swelling curve, swelling parts of the swelling curve, and swelling-collapse curve, curves were approximately linear in the e-lns plane. The initial swelling index is constant and depends on the OCR. Therefore, the relationship between the initial swelling indexes with the OCR could be obtained and is presented in Figure 10. Figure 10 shows that the larger the OCR (the farther the distance between stress state and LC curve), the larger the index of expansion. When OCR decreased to 1, the stress state reached the LC curve and $\lambda_{s}^{*}$ decreased to 0. No more swelling strain can be generated.

The above analysis shows that the swelling index of bentonite is determined by two parameters: suction and over-consolidation ratio, which can be expressed as,

(2) $$\lambda_{s}=\mu {\left(\ln\frac{p_{c}}{p}\right)}^{\xi }\exp\left(-s\right)+\eta \ln\frac{p_{c}}{p}$$

where, $\lambda_{s}$ is the swelling index. *p* and *p*_c_ are applied stress and yield stress, respectively. μ, ξ, η are soil parameters.

(3) $${d\varepsilon }_{v,s}^{e}=\frac{\kappa_{s}}{1+e_{0}}\frac{ds}{s}$$

(4) $${d\varepsilon }_{v,s}^{p}=\frac{\lambda_{s}-\kappa_{s}}{1+e_{0}}\frac{ds}{s}$$

where, ${d\varepsilon }_{v,s}^{e}$ and ${d\varepsilon }_{v,s}^{p}$ are elastic and plastic swelling strain increment caused by s, respectively. $\kappa_{s}$ is the slope of the reversible wetting–drying line in an e-ln(s) diagram.

For the collapse part on the void ratio–suction (e-lns) curves, collapse strain could be described by the method which is the same with BBM. The LC yielding curve [21,22] is expressed by Equation (5).

$${d\varepsilon }_{v,s}^{p}={d\varepsilon }_{v,p}^{p}=\frac{\lambda \left(0\right)-\kappa }{1+e_{0}}\frac{dp_{0}^{*}}{p_{0}^{*}}$$

with

(5) $$\lambda \left(s\right)=\lambda \left(0\right)\left[r+\left(1-r\right)e^{-\beta s}\right]$$

where, λ(s) and λ(0) are the slope of the virgin consolidation line at suction s and saturated state, respectively. $p_{0}^{*}$ and $p_{c}$ are the saturated pre-consolidation pressure and reference stress, respectively. r and β are model parameters.

**Figure 11:**
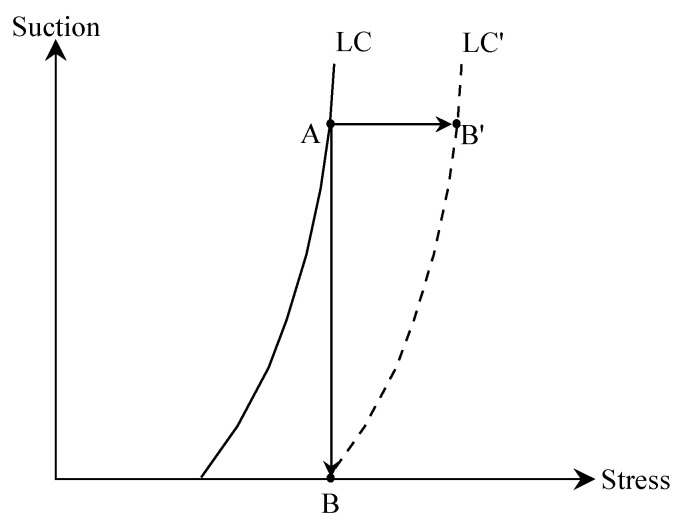
The collapse strain induced by the suction-decrease path. AB: suction reduction path, AB′: loading path.

As shown in Figure 11, when suction decreased from A to B, the bentonite was in the yielding state, and collapse strain was generated instead of the swelling one. Meanwhile, the LC yielding curve moved to LC′ due to hardening. It could be found that the hardenings (LC movement) during the suction reduction path AB and loading path AB′ were the same, and thus the plastic shrinkage strains were the same. Therefore, the collapse strain induced by decreasing s can be expressed by,

(6) $${d\varepsilon }_{v,s}^{p}={d\varepsilon }_{v,p}^{p}=\frac{\lambda \left(0\right)-\kappa }{1+e_{0}}\frac{dp_{0}^{*}}{p_{0}^{*}}$$

where, ${d\varepsilon }_{v,p}^{p}$ is plastic strain increment caused by p. κ is the slope of the reversible loading–unloading line in an e-lnp diagram.

For assessing the performance of the proposed equation of void ratio–suction curves, the suction-controlled swelling deformation tests under constant vertical loads carried out on compacted GMZ bentonite specimens in this work were simulated. For GMZ bentonite, parameters of the proposed equation were determined and are listed in Table 2.

**Figure 12:**
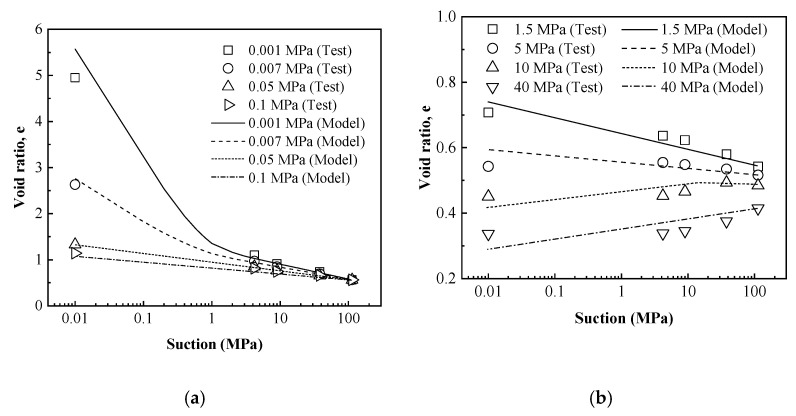
Simulations of swelling deformation tests conducted on compacted GMZ bentonite. (**a**) 0.001~0.1 MPa. (**b**) 1.5~40 MPa.

Simulated results obtained by the proposed equations were compared to the experimental data in Figure 12. Results in Figure 12 show that the volume change behavior under hydration could be well described by the proposed equations, especially in terms of the different types of the hydration deformation curves.

## 5. Conclusions

In this work, suction-controlled swelling deformation tests under constant vertical stresses were carried out on compacted GMZ bentonite specimens. The following conclusions were drawn.

The void ratio–suction curves during hydration presented four typical shapes. The different swelling-collapse behavior during hydration depends on suction and OCR. Based on this, the swelling index was defined. The equations for describing the swelling-collapse characteristic of void ratio–suction curves were proposed. Simulation results of suction-controlled swelling deformation tests showed that the different types of the hydration deformation curves could be well described by the proposed equations. The results can provide significant mechanical parameters for successful design and construction of engineered barriers. Moreover, equations are proposed for description and prediction of swelling characteristic of compacted bentonite during hydration, which are also of great importance for the safety assessment of the HLW repositories.

## Figures and Tables

**Table 1:** Specifications of the swelling deformation tests.

Tests	Vertical Stress during Saturation (MPa)	Suction Paths (MPa)
1	0.001	113-38-9-4.2-0.01
2	0.007	113-38-9-4.2-0.01
3	0.05	113-38-9-4.2-0.01
4	0.1	113-38-9-4.2-0.01
5	1.5	113-38-9-4.2-0.01
6	5	113-38-9-4.2-0.01
7	10	113-38-9-4.2-0.01
8	40	113-38-9-4.2-0.01

**Table 2:** Parameters for GMZ bentonite.

*λ*(0)	*κ*	$p_{0}^{*}$	*r*	*β*	*κ_s_*	*μ*	*ξ*	*η*
0.15	0.01	5.3	0.852	0.1004	0.001	9.274 × 10^−7^	6.037	0.0149

## Data Availability

All data, models, or code that support the findings of this study are available from the corresponding author.
